# Books

**Published:** 1883-03

**Authors:** 


### Books.

---

A Manual of Gynecology. By D. Berry Hart, M.D., F.R.C.P E., and A. H. Barbour, M.A., B.Sc., M.B. Volume 1, with Eight Plates and One Hundred and Ninety-two Wood Cuts. New York: William Wood & Co. 1883. Wood's Library of Standard Medical Authors.

This volume is subdivided into six sections, which treat respectively of the anatomy and physiology of the female pelvic organs; exploration of the pelvic organs by the hands and instruments; affections of the peritoneum and connective tissue; of the fallopian tubes and ovaries; and of the uterus.

In scanning the pages of this work one cannot fail to be impressed with the important contributions made to this department of medical science by American investigators; well-known names, inseparable from the history of this important and progressive line of study, being of frequent recurrence.

Chapter xix is devoted exclusively to "Battey's operation."

"The real history of this operation," say the authors, "dates from August 17, 1872, when Battey, of Rome, Ga., U. S. A., successfully removed the ovaries of a patient who suffered from intolerable dysmenorrhœa. On July 27, of the same year, Hegar, of Freiburg, removed both ovaries in a case of severe ovarian neuralgia; the patient died and Hegar did not publish an account of the case. To Battey, however, is due the honor of independently conceiving the idea and—what was more difficult—of successfully carrying it into execution and impressing the profession with its importance and value in special cases.

"We have adopted the term 'Battey's operation,' first proposed by Marion Sims, as a convenient and useful one. Other terms, however, have been proposed. The name 'normal ovariotomy' is a misnomer inasmuch as the ovaries are not normal. 'Spaying,' a term recommended by Goodell, does not recommend itself by its delicacy."

The nature and aims of the operation, and the indications for its use and its results are then described and finally it is concluded that "This operation is as yet on its trial. Gynecologists have not yet settled the exact indications for it, nor the question as to whether it is always worth the risk."

This volume is the initial number of the series for the present year—which, by the way, promises to surpass any of the preceding sets—and the superior appearance and fine workmanship will, no doubt, tend to enhance the deserved popularity of these valuable libraries of standard works. The illustrations are numerous and creditably executed.

---

THE HOSPITAL TREATMENT OF DISEASES OF THE HEART AND LUNGS, with over Three Hundred and Fifty Formulæ and Prescriptions as Exemplified in the Hospitals of New York City. By Charles H. Goodwin, M.D. New York: C. H. Goodwin, M.D. 1883. Pp. 196. Price, Cloth, $1.50.

This little work contains a resume of the treatment of some of the more important and more common diseases of the heart and lungs as exemplified in the practice of specially capable and skilful practitioners in the various hospitals of New York city. It is modest in its pretentions and doubtless honest in its representations. It is calculated to accomplish good in its way, and, in the comparatively narrow field which it seeks to occupy, we wish it the success which we believe it deserves.

The names of such men as Alonzo Clark, Austin Flint, Alfred L. Loomis, A. C. Post, Beverly Robinson, A. Jacobi and others of note and merit, as the authors of the doctrines herein set forth, certainly commend the little book to confidence.

---

POCKET THERAPEUTICS AND DOSE BOOK. By Morse Stewart, Jr., B.A., M.D. Third Edition Revised and Enlarged. Detroit, Michigan. Geo. D. Stewart & Co. 1882. Pp. 240. Price, Cloth, $1.00, Morocco $1.50.

This neat little volume is dedicated to the consideration, very briefly, of such subjects as prescription writing; list of abbreviations used in prescriptions, table of doses for various ages, genitive endings, number of drops to the drachm of different fluids, weights and measures, classification and explanation of the action of medicines, and the several modes of introduction, posological and therapeutic table, formulas, emergencies, poisons and antidotes, etc., etc.

The contents are judiciusly selected and conveniently arranged. The little book is clear, compact and comprehensive, and is one of the very best of its kind.

---

A Manual of Histology. Edited and Prepared by Thomas E. Satterwaite, M.D., of New York, in Association with Drs. Thomas Dwight J. Collins Warren, William F. Whitney, Clarence J. Blake, and C. H. Williams, of Boston; Dr. J. Henry C. Simes, of Philadelphia; Dr. Benjamin F. Westbrook, of Brooklyn; and Drs. Edmund C. Wendt, Abraham Mayer, R. W. Amidon, A. R. Robinson, W. R. Birdsall, D. Beyson Delavan, C. L. Dana and W. H. Porter, of New York. Second Edition, Enlarged and Revised, Containing Two Hundred and Two Illustrations, with an Appendix. New York: William Wood & Co. 1882. Pp. 490.

Only a few months ago we had the pleasure of offering some favorable comments upon this work, the popularity of which has rendered, at this early day, a second edition necessary.

The text has not been changed except to correct typographical and other unavoidable and unimportant errors, but such progress as has been made in histological investigations since the publication of the first edition are noted in the appendix. We can conscientiously commend the book.

---

Transactions of the Medical Society of the State of Pennsylvania at its Thirty-Third Annual Session, Held at Titusville, May 10, 11 and 12, 1882. William Varian, M.D., Titusville, President; William B. Atkinson, M.D., Philadelphia, Secretary.

This is a handsome cloth-bound volume of four hundred and sixty-seven pages and gives proof of the healthy state of the organization whence it emanates. The treasurer's report shows a balance of two thousand dollars in the treasury after paying all expenses of the preceding year. The several addresses and the papers presented at the meeting afford interesting and instructive reading.

---

THE TRANSACTIONS OF THE MEDICAL ASSOCIATION OF THE STATE OF MISSOURI AT ITS TWENTY-FIFTH ANNUAL SESSION, HELD AT HANNIBAL, MAY, 16, 17 AND 18, 1882. A. E. Gore, M.D., Paris, President; C. A. Todd, M.D., St. Louis, and J. H. Duncan, Columbia, Secretaries.

This is a neatly arranged and well printed volume, in pamphlet form, of two hundred and twenty pages, and contains, besides the record of this meeting, lists of members, etc., a fair assortment of papers on topics of interest to the profession. The president's address is devoted to "Quacks and Quackery in Missouri" and clearly establishes the justice of the claim of this State to the palm in this sort of villainy. It furnishes a strong argument in favor of stringent and wisely conceived laws for the regulation of the practice of medicine, and for the protection of the people from this dangerous and aggressive element. An excellent feature of this volume is the report and publication of the discussions upon the papers read before the society. This practice materially increases the value of and the interest in the proceedings.

---

THE MEDICAL SOCIETY OF THE STATE OF TENNESSEE, TRANSACTIONS, 1882. FORTY-NINTH ANNUAL MEETING. W. F. Glenn, M.D., Nashville, President; C. C. Fite, M.D., Nashville, Secretary.

It is refreshing in taking up a volume of society transactions to find that its contents are not monopolized by the effusions of psendo-specialists, who, disregardful of the real objects and true intrests of such organizations, insist upon proclaiming themselves and their peculiar fitness for special practice, and, by a public announcement of the number of operations performed, the number of cures effected, etc., to promote their own material advancement unmindful of the claims of science, and of the proprieties of such occasions.

In this volume of 186 pages the contributions are such as to interest and instruct the practical physician, and are free from the objectionable features alluded to above.

